# Rising Publication Delays Inflate Journal Impact Factors

**DOI:** 10.1371/journal.pone.0053374

**Published:** 2012-12-31

**Authors:** Adriano B. L. Tort, Zé H. Targino, Olavo B. Amaral

**Affiliations:** 1 Brain Institute, Federal University of Rio Grande do Norte, Natal, Brazil; 2 Institute of Medical Biochemistry, Federal University of Rio de Janeiro, Rio de Janeiro, Brazil; University of Illinois-Chicago, United States of America

## Abstract

Journal impact factors have become an important criterion to judge the quality of scientific publications over the years, influencing the evaluation of institutions and individual researchers worldwide. However, they are also subject to a number of criticisms. Here we point out that the calculation of a journal’s impact factor is mainly based on the date of publication of its articles in print form, despite the fact that most journals now make their articles available online before that date. We analyze 61 neuroscience journals and show that delays between online and print publication of articles increased steadily over the last decade. Importantly, such a practice varies widely among journals, as some of them have no delays, while for others this period is longer than a year. Using a modified impact factor based on online rather than print publication dates, we demonstrate that online-to-print delays can artificially raise a journal’s impact factor, and that this inflation is greater for longer publication lags. We also show that correcting the effect of publication delay on impact factors changes journal rankings based on this metric. We thus suggest that indexing of articles in citation databases and calculation of citation metrics should be based on the date of an article’s online appearance, rather than on that of its publication in print.

## Introduction

The impact factor was first introduced in 1972 by Eugene Garfield [1] and has been used as a primary tool for evaluating the quality of scientific publications ever since. It is defined as the number of citations in a given calendar year to articles published in a journal over the two preceding years, divided by the total number of citable articles published by the journal in the same period. Although overreliance on impact factors has been widely criticized, both for the metric’s inherent limitations [2], [3] and for fostering an obsession with “high-impact” science [4], evidence shows that they are highly correlated with perceived journal quality [5] and influence the evaluation of institutions and individual researchers worldwide [6]. For these reasons, scientific publications take active steps to increase and publicize their impact factors. These steps can sometimes include the use of dubious means, such as selective publication of highly cited types of articles, self-citations or coercion of authors to cite the journal [7], [8].

**Figure 1:**
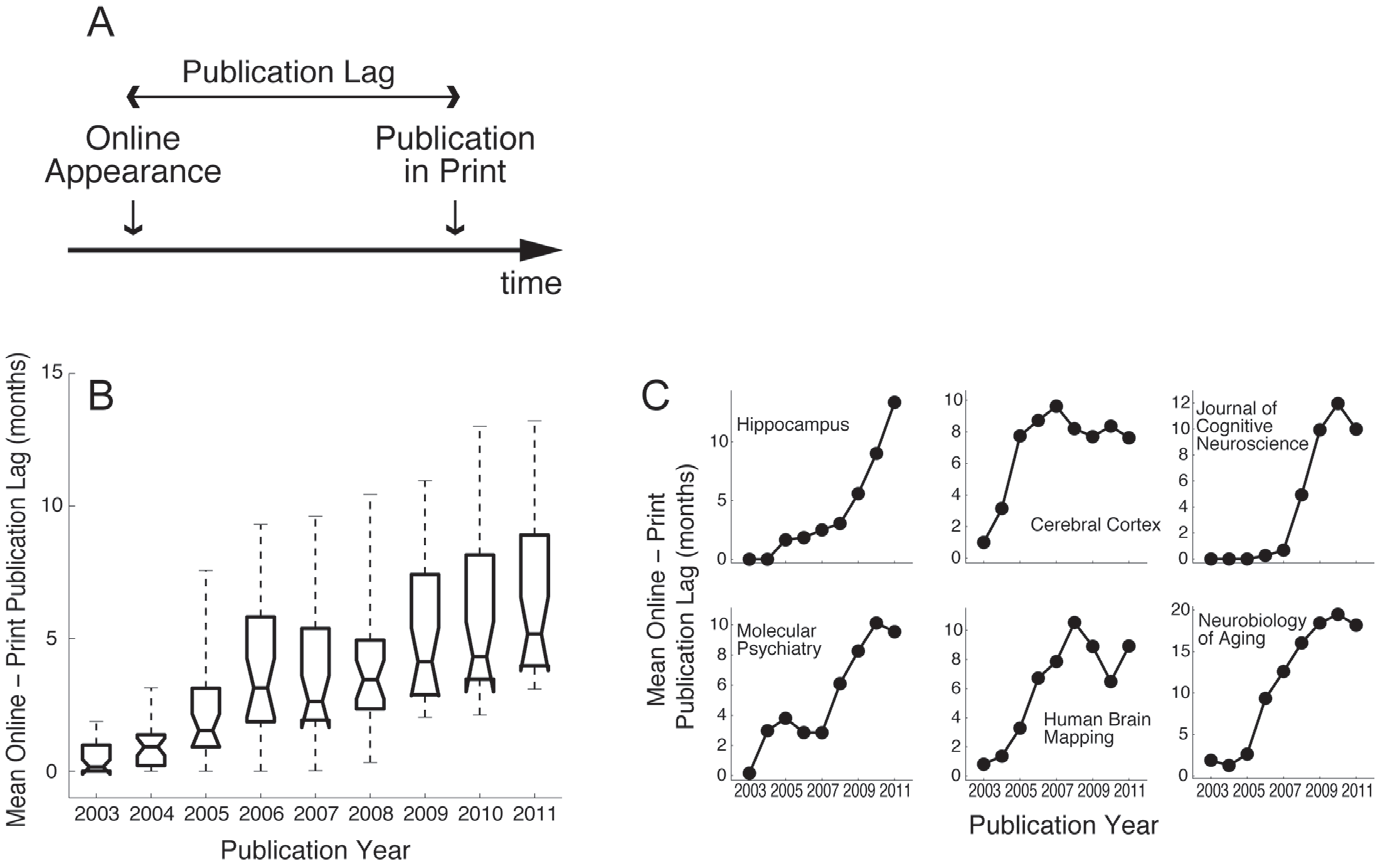
Increase in online-to-print publication lags from 2003 to 2011. (A) Schematic depiction of the online-to-print lag. The publication lag is defined as the time period between the date of the online appearance of an article and that of its official publication in print. During the publication lag, articles are usually categorized as “in press”, “early view”, or “ahead of print”. Impact factors are calculated based upon an article’s publication in print. (B) Distribution of online-to-print lags over the years for all 31 neuroscience journals with lags greater than 3 months in 2011. Boxes indicate the median, 25^th^ and 75^th^ percentiles; whiskers indicate the 5^th^ and 95^th^ percentiles. (C) Mean online-to-print lags as a function of publication year for 6 neuroscience journals exhibiting steep increases in this measure.

The concept of publication delay traditionally refers to the time between the acceptance of an article and its publication and indexing in scientific databases. It is normally viewed as a problematic issue, and has been previously proposed to correlate negatively with journal impact factors [9]. With the advent of online access, however, electronic publishing of articles in preliminary or final form before print publication and indexing has become commonplace. Thus, a significant fraction of publication delay now consists of a period in which an article is available online, but has not been formally published in print. We will refer to this period, illustrated in Figure 1A, as “online-to-print lag”.

Despite the fact that many articles are now available online before they are published in print form, the Institute for Scientific Information (ISI) (http://www.isinet.com) database, on which impact factor calculations are based, still indexes articles only upon their “official” publication date; this date, with a few exceptions (most notably the case of primarily electronic journals), usually corresponds to the date of an article’s appearance in print. As most articles are now primarily accessed and read online [10]–[12], however, this could lead to an interesting phenomenon: since papers which are available as “in press” online can be read and cited promptly, they might have a greater chance of being cited during the 2-year window upon which impact factors are based, compared to articles with no online-to-print lag. Increased citation rates have already been shown for papers posted in preprint archives before publication in areas of science where this practice is common (the “early access” effect) [13]–[15]. Thus, we hypothesized that the existence of online-to-print lags might artificially inflate journal impact factors.

In this study, we used publication records of neuroscience journals to analyze the evolution of publication delay over the last decade, and to study whether this phenomenon can alter journal impact factors. We show that online-to-print lags have risen steeply in recent years, and that their existence leads to impact factor inflation. Furthermore, we show that this effect is greater for journals with larger online-to-print lags, and that this fact can influence journal rankings based on impact factors.

## Methods

### Journal Selection

We first considered all 107 journals under the “Neurosciences” category in the Journal Citation Report (JCR) database published by ISI (http://webofknowledge.com/JCR) that had a 2010 impact factor greater than 3. We then excluded 46 journals from this list either because of (*a*) low number of papers published (we only analyzed journals that published >60 papers in 2010), (*b*) lack of consistent impact factor data given the recency of the journal’s indexing, (*c*) low publication frequency (we only considered journals that had at least 8 issues per year), or (*d*) lack of information about publication month. Exclusion criteria *a* and *b* were required because the impact factor of these journals suffers considerable fluctuations across years, making it difficult to isolate the effect of publication lags from other factors and/or random variation. We adopted exclusion criteria *c* and *d* because the time resolution of our analysis was in the order of months; thus, journals with low periodicity or lack of information on publication month could not be analyzed at sufficient resolution. The list of the 61 journals selected for further analysis is available in Table S1.

### Data Sources and Analysis

Citation counts were obtained from the ISI Web of Knowledge website (http://webofknowledge.com). Dates of online appearance and print publication were obtained from individual article entries in PubMed (http://www.ncbi.nlm.nih.gov/pubmed). All data analysis was performed in Matlab (Mathworks, Inc.), and the datasets used for analysis are available as Data S1.

### Estimation of Online-to-print Lags for Selected Journals

Online-to-print lag was computed as the difference between the online appearance date of an article and its official date of publication, as obtained from PubMed records. Mean online-to-print lags for journals were obtained from all published articles in a given calendar year.

### Estimation of 2010 Impact Factors

The 2010 impact factor is defined as the number of citations in 2010 to articles published in 2008 and 2009 divided by the number of articles published in 2008 and 2009. In this work, we estimated journal impact factors by taking into account only articles and reviews (both in the numerator and denominator of the impact factor formula). Note that this is not identical to the procedure used by ISI, which estimates impact factors by dividing the total number of citations to a journal by the total number of “citable items", which varies according to the individual formats published by each journal [3]. Moreover, ISI considers all citations to a journal, irrespectively of whether or not the precise identity of the article being cited can be retrieved (see also Discussion). This was not the case in our calculations, which tracked citations linked to individual indexed articles, since all of our analyses depended on article publication dates. Thus, by not considering unlinked citations, our estimation of impact factors systematically provides lower values than those reported by ISI. Importantly, we note that the unlinked citations tracked by ISI only increase the numerator of its impact factor formula (number of citations) without changing the denominator (number of articles) [16]; in the Discussion section, we comment on how this feature can also contribute to the inflation of impact factors brought about by online-to-print lags.

### Estimation of the Lag-corrected 2010 Impact Factor Index

**Figure 2:**
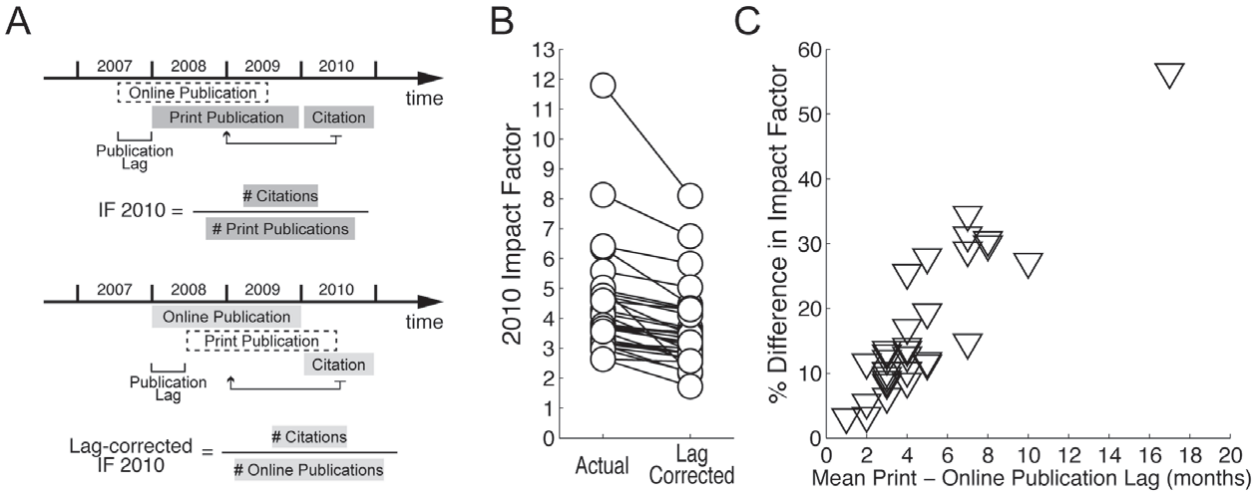
Online-to-print publication lags inflate impact factors. (A) Schematic representation of the lag-corrected impact factor index. The 2010 impact factor (*top*) is calculated by considering the number of citations in 2010 to articles officially published (i.e. in print) in 2008 and 2009, divided by the number of articles officially published in 2008 and 2009. The lag-corrected 2010 impact factor (*bottom*) considers the number of citations in 2010 to articles that were published online in 2008 and 2009, divided by the number of articles published online in 2008 and 2009. (B) Actual and lag-corrected impact factors for the same 31 journals as in Figure 1B. Lag-corrected impact factor is smaller than the actual one for all journals (p<10^−5^, paired t-test). (C) Scatter plot showing a strong correlation between the difference between actual and corrected impact factors (in %) and the duration of the publication lag (r = 0.90, p<10^−11^).

To calculate the lag-corrected impact factor index, we considered citations in 2010 to articles published in a two-year time window, shifted from 2008–2009 to a later time point by the mean online-to-print publication lag of the journal in 2008–2009. To obtain the lag-corrected impact factor for each journal, we divided the total amount of 2010 citations to articles published in the shifted two-year window by the number of articles in the same period (see Figure 2A). For example, in the case of a 3-month lag, we considered papers published between April 2008 and March 2010, which would have appeared online between January 2008 and December 2009. We also repeated the same analysis after exclusion of articles cited more than 2 standard deviations above the mean citation rate of their respective journals, in order to evaluate the effect of these outliers on impact factor inflation.

### Estimation of 2010 Impact Factors for Simulated Lags

Only journals with short publication lags (<1 month) were taken into account in this analysis, in order to contrast a situation where no significant lag was present to those in which online-to-print lags of various durations were simulated. Since neuroscience journals with short lags were infrequent (only 13 were found, or ∼21% of the total), in this analysis we also included 12 general scientific journals that publish neuroscience research and have short (<1 month) online-to-print lags: *Biophysical Journal, Cell, Current Biology, Nature, Nature Biotechnology, Nature Cell Biology, Nature Methods, PLoS Biology, PLoS Computational Biology, PLoS ONE, Proceedings of the National Academy of Sciences* and *Science.*

**Figure 3:**
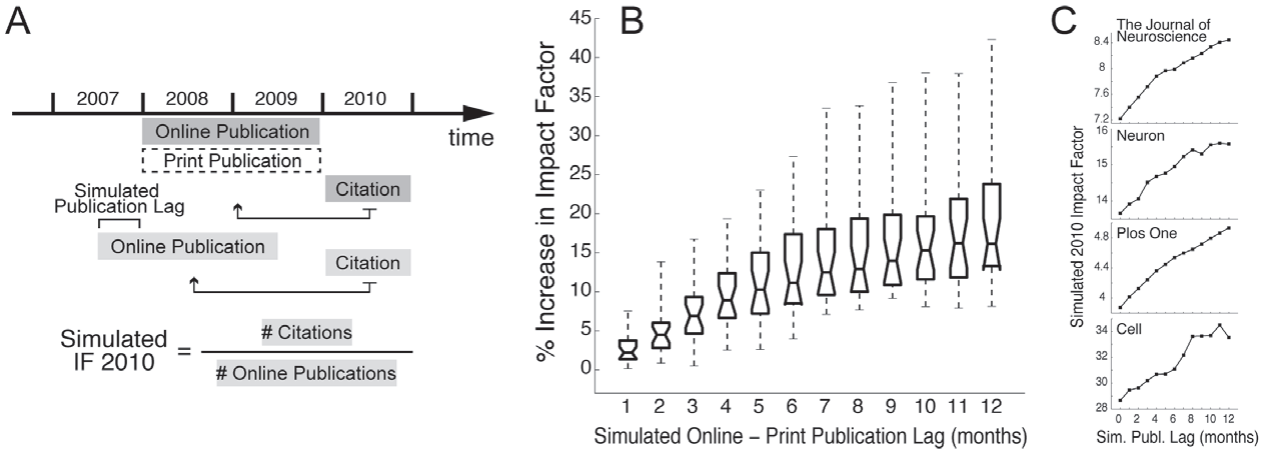
Journal impact factors positively correlate with the duration of online-to-print publication lags. (A) Schematic depiction of the estimation of 2010 impact factors for simulated lags. To simulate the effect of online-to-print lags on journal impact factors, the number of citations in 2010 to articles published in a 2-year window, shifted from 2008–2009 by the length of the simulated lag, is divided by the number of articles published in that period. (B) Percentage increase in impact factors as a function of the duration of simulated publication lags (left). This result was obtained by simulating online-to-print lags in 25 journals with negligible publication lags (<1 month). Boxes and whiskers represent percentiles as in Figure 1B. (C) Individual examples of the positive relation between a journal impact factor and the duration of the publication lag for 4 journals with negligible or nonexistent lags.

In order to simulate an online-to-print lag of *n* months, we considered citations in 2010 to articles published in a two-year window, shifted to an earlier time point from 2008–2009 by *n* months. To obtain the impact factor with the simulated lag, we divided the total number of 2010 citations to articles in this window by the number of papers in the same period (see Figure 3A). The rationale for this algorithm is as follows: the 2010 impact factor is defined as the number of citations in 2010 to the articles published by a journal in 2008 and 2009, divided by the total number of articles in 2008–2009. A publication lag of 6 months means that articles that appeared in print between January 2008 and December 2009 would have actually appeared online between July 2007 and June 2009. Therefore, in order to simulate the effect of a 6-month lag in a journal that has no publication lag (i.e., in which the date of online and print publication is the same), we considered citations in 2010 to articles published between July 2007 and June 2009, divided by the number of articles in this time window. We consider that, if a 6-month publication lag was present, these papers would have been indexed as officially published between January 2008 and December 2009, and thus would be the ones contributing to the 2010 impact factor. In this way, we can simulate the effect of a 6-month online availability period prior to publication of articles of this journal, similarly to what happens with journals that have actual 6-month delays.

## Results

After filtering by the criteria described in the Methods section, we analyzed 61 neuroscience journals indexed by ISI (Table S1). Calculations of online-to-print lags from 2003 to 2011 showed that these lags have risen markedly over the last 10 years. Whereas this phenomenon was virtually nonexistent for most journals in 2003, around 50% (31/61) of the journals in our sample currently have online-to-print lags of more than 3 months, with a mean of 6.4 months in 2011 (Figure 1B). Importantly, the size of these lags varied widely among journals, ranging from 0 to 18 months. Figure 1C shows examples of the evolution of online-to-print lags for journals with large lags in 2011.

As discussed in the introduction, large online-to-print lags may increase the chances that an article is cited in the 2-year window used to calculate impact factors, raising the possibility that they may artificially increase the impact factor of some journals. To study whether this inflation of impact factors actually occurs, we calculated lag-corrected impact factor indexes based on the online publication date, as illustrated in Figure 2A. We found that the lag-corrected impact factor was smaller than the one based on print publication for all of the 31 neuroscience journals with online-to-print lag >3 months (p<10^−5^, paired t-test) (Figure 2B and Table S2). More importantly, the relative size of this difference (in %) correlated strongly with the duration of the publication lag (r = 0.90, p<10^−11^) (Figure 2C). This effect persisted after excluding articles cited more than 2 standard deviations above the mean (Figure S1), and its relative size was independent of the original impact factor of the journal (Figure S2), showing that the inflation of impact factors was not driven only by highly cited papers. Thus, it appears that a large online-to-print publication lag can indeed inflate a journal’s impact factor artificially.

While this effect is highly significant, it could be attributed to the fact that citations occurring before print publication (i.e. within the publication lag) are not considered in our analysis – while they do count for the ISI impact factors, albeit only in the numerator, as described in the Methods section and in [16]. Therefore, computing impact factors based on the online publishing date will not detect citations occurring in this period, potentially biasing the corrected impact factor index, particularly for journals with very long lags. Since the lack of information on preprint citations to articles before they are indexed in the ISI database prevents us from correcting this bias, we simulated the effect of delaying print publication of all 13 journals in our sample (∼21%) with short (<1 month) online-to-print lags, as well as of 12 general scientific journals that also publish neuroscience research with negligible lags, as shown in Figure 3A. As expected, we found that impact factors rose proportionally to the increase in online-to-print publication lag, with a mean increase of ∼20% after 12 months (range: 8–47%; Figure 3B). Examples of this effect in the case of specific journals are shown in Figure 3C. Importantly, in this case the results cannot be attributed to uncounted citations. One should note that our simulations are based on the premise that articles will be equally cited when available online, whether published in print or not, which is probably a valid assumption considering current patterns of article searching by scientists [12], as well as the fact that articles available online have much higher citation rates than those only available in print [17].

**Figure 4:**
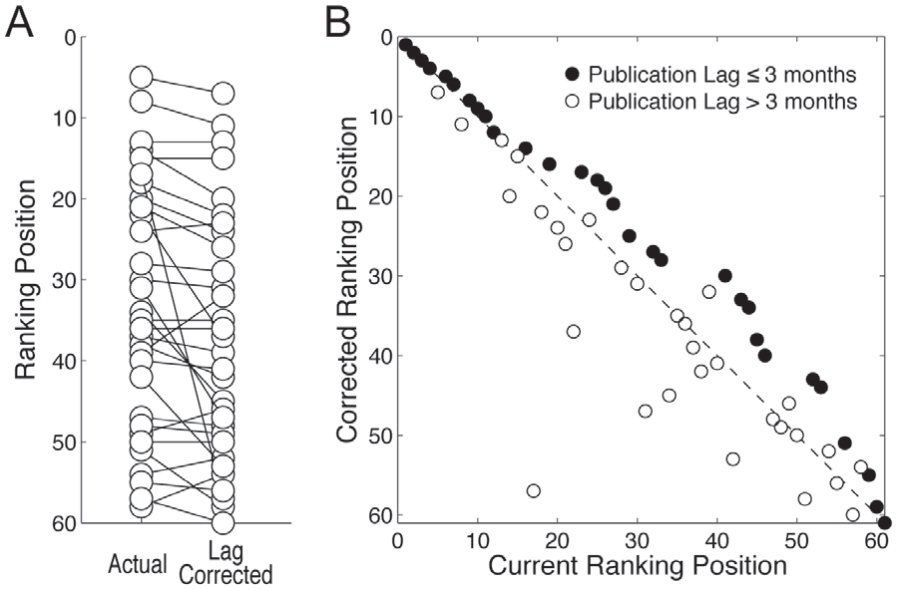
Online-to-print publication lags influence journal rankings based on impact factors. (A) Impact factor ranking position for 31 neuroscience journals with publication lag >3 months before and after lag correction. (B) Lag-corrected vs current ranking position for 61 neuroscience journals. Rankings tend to increase for journals with publication lags ≤3 months (black circles), and to decrease for journals with publication lags >3 months (white circles). Note that part of these changes could be attributed to the bias related to uncounted citations, especially for journals with very long online-to-print lags (see Results).

Finally, we found that the artificial inflation of impact factors resulting from online-to-print lags can influence ISI journal rankings. This is shown in Figure 4 and Table S2, which depict the changes in impact factor rankings among the 61 journals for the 31 journals with lags greater than 3 months. As expected, journals with long publication lags showed sharp decreases in ranking after correction of the impact factor. We note, however, that part of this decrease, especially in the case of very long online-to-print delays, could be attributed to the bias related to uncounted citations occurring during the lag period, as described above.

## Discussion

Our results show that the delay between online availability of articles and print publication has been steadily increasing over the last decade, and that this can have significant effects on journal impact factors. Online availability of articles in preprint archives had already been shown to increase early citation rates in areas of science such as astrophysics and astronomy [14], [15] – however, as preprint archiving of articles is a decision of authors, one could not fully exclude a selection bias effect in this case [13], [15]. By concentrating on journals instead of individual articles, our results provide the clearest demonstration up to date that online availability before print publication leads to earlier citations, and thus to an increase in impact factors.

We believe these findings have important implications. The first is to call attention to the fact that most of the metrics currently used for the evaluation of scientific output, including impact factors, were designed for a system based on print publication that no longer exists, as the vast majority of articles are now accessed and read online [10]–[12]. This can lead to important distortions, with concrete effects on policy decisions concerning the evaluation of journals and/or individual scientists. A reformulation of these measures is therefore urgent, and both the indexing of articles on the date of online publication and the use of metrics based on this date seem to be warranted.

A second and subtler implication concerns the reasons for the steep rise in publication lags over the years. This phenomenon could represent just a casual consequence of the increase in the agility of online publication – as online publishing becomes easier and print publication remains equally slow, it is natural to expect that the demand for online availability could lead to an increase in online-to-print lags. Moreover, increased lags could also be due to the sheer increase in the numbers of papers submitted and published over the years. However, many ways of “playing the system” by journal editors in order to increase impact factors have been described in the past, such as selectivity in publication formats and coercion of authors to include citations to the same journal [7], [8]. Thus, it is at least feasible that increasing online-to-print lags might represent an active editorial policy to try to raise impact factors in some cases.

We have demonstrated that the longer a paper is available as in press, the higher are its chances of getting cited in the 2-year window after it is published in print (presumably due to the fact that papers available online can be read and used by scientists more promptly and are thus cited earlier after publication), leading to an increase in the impact factor of journals with long online-to-print lags. In addition to this effect, we note that there is a second mechanism by which publication delays can inflate the ISI impact factors published in JCR. This is the fact that citations to articles while in press only contribute to the numerator of the impact factor formula (number of citations), without changing its denominator (number of articles) [16]. This occurs because the numerator of the ISI impact factor tracks all citations to a journal, irrespective if the cited item was indexed or not (see Methods); however, only indexed articles count for the denominator of the impact factor formula employed by ISI. Unfortunately, we are not able to measure this effect from the data provided in the ISI Web of Science, as citations to in press articles that have not been indexed cannot be retrieved in a systematic way. Nevertheless, it is likely that both effects interact, leading to an even larger inflation of impact factors than the one we have described.

We illustrate these effects by the following example: suppose an article is published online in 2008, and officially published and indexed in 2009. Until its official publication, citations in 2009 to this article while in press will count as citations to a 2008 item of its journal; this will inflate the journal's 2009 impact factor, since citations to this article will increase the numerator of the 2009 impact factor formula (in this case, number of citations in 2009 to 2007 and 2008 "citable items", see Methods), while this work will not be counted in the denominator (number of papers indexed in 2007 and 2008). After the article is officially published, it is then considered as a 2009 publication, and will be included in the estimation of 2010 and 2011 impact factors. From then on, the inflation of impact factors will be brought about by the fact that citations in 2010 will be more numerous for this paper than for a similar one published both online and in print in 2009, as this paper will have been available online to be read and used since 2008.

Taking all of this into account, we believe our results raise the issue that overreliance on very specific metrics such as impact factors should be taken with care, as they can be prone to distortion and/or manipulation. In this sense, alternative ways to measure the impact of journals or articles irrespectively of citations (brought about by the plethora of information which can be drawn from online access data) have been proposed recently, such as online usage metrics [18], network-based statistics [19], [20] and replicability tracking [21]. Although our data do not constitute evidence against using citations to evaluate impact, they do suggest that more complex forms of assessing journal quality should be sought, as the use of multiple measures in scientometrics will almost inevitably be more representative and less prone to distortion than reliance on a single index.

Moreover, our data call for a reformulation of citation records by existing databases. Not only for calculation of impact factors, but for any measure that includes citations within a specific time window, we believe that it is more appropriate to use databases that index articles on the basis of the date when they are first available online. Although in the long run simple citation-based metrics such as impact factors are likely to give way to more complex metrics, we acknowledge that they still play an important role in evaluating journal quality at the present time. In a world that has been revolutionized by electronic publication, it is important to discuss how to reform these traditional tools for evaluating scientific impact in order for them to maintain their relevance. In this sense, a simple measure to avoid distortions such as the one described here is the indexing of articles by scientific databases on the date of their online appearance, rather than on that of their publication in print.

## Supporting Information

Figure S1 Inflation of impact factors by online-to-print lags is not exclusively determined by highly cited articles. (A) Actual and lag-corrected impact factors for the same 31 journals as in Figure 2B after removal of articles cited more than 2 standard deviations above the mean citation rate for each journal. A similar decrease in impact factor after lag correction is still observed for all journals (p<10^−7^, paired t-test). (B) Scatter plot showing that the correlation between the decrease in impact factor caused by lag correction (in %) and the duration of the publication lag remains strong after removal of these outliers (r = 0.91, p<10^−11^). (C) Scatter plot showing the % difference in impact factor caused by lag correction for each journal, both before (*x* axis) and after (*y* axis) exclusion of outliers. There is a slight trend for decrease in the lag-corrected difference after outliers are removed, but this does not reach statistical significance (p = 0.12, paired t*-*test).
(TIF)

Figure S2 Online-to-print publication lags and inflation of impact factors occur independently of the original journal impact factor. (A) Scatter plot showing absence of significant correlation between journal impact factor and mean publication lag for the 31 journals with lags longer than 3 months (r = 0.20, p = 0.28). (B) Scatter plot showing absence of significant correlation between journal impact factor and relative difference in impact factor after lag correction (r = 0.23, p = 0.21).
(TIF)

Table S1 Journal list.
(PDF)

Table S2 Lag-corrected impact factors and journal rankings.
(PDF)

Data S1 Datasets used for the analysis.
(ZIP)
