# Supplementary figures and images for: Rising Publication Delays Inflate Journal Impact Factors
Source: PLoS One. 2012 Dec 31;7(12):e53374. doi: 10.1371/journal.pone.0053374 (PMC3534064; doi:10.1371/journal.pone.0053374)

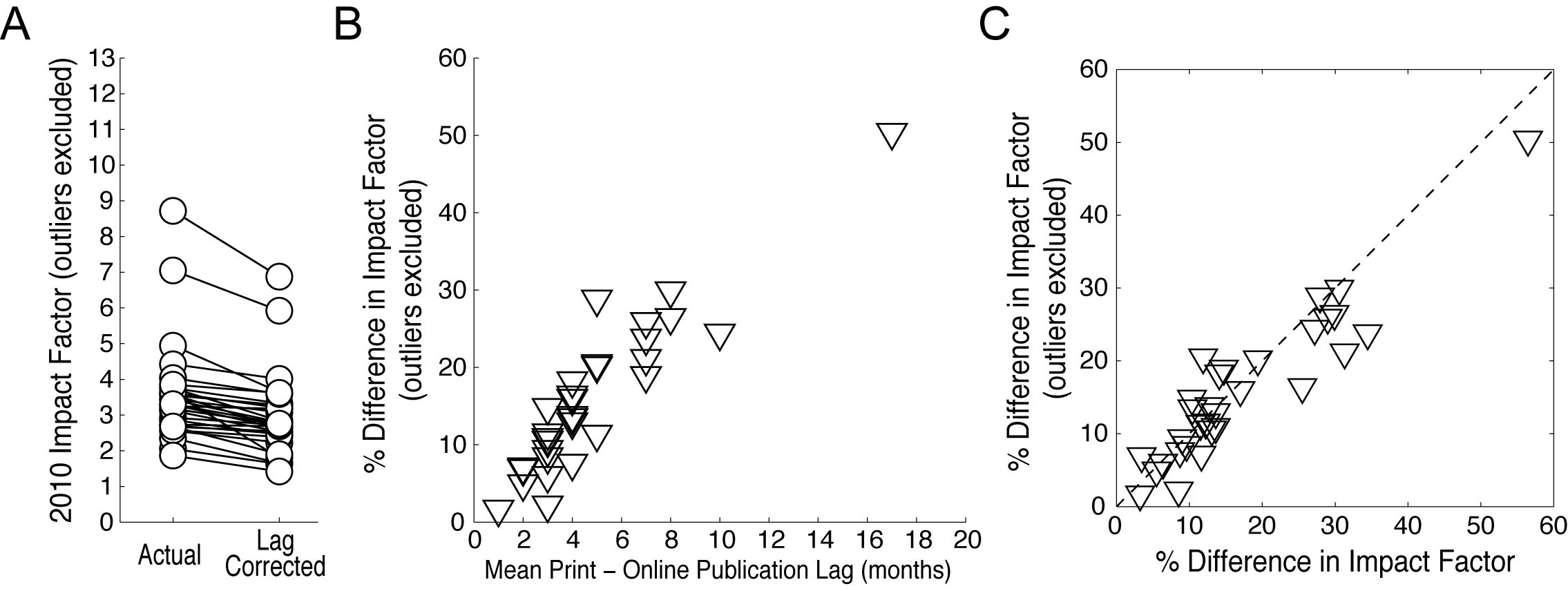

Supplement: Figure S1 — Inflation of impact factors by online-to-print lags is not exclusively determined by highly cited articles. (A) Actual and lag-corrected impact factors for the same 31 journals as in Figure 2B after removal of articles cited more than 2 standard deviations above the mean citation rate for each journal. A similar decrease in impact factor after lag correction is still observed for all journals (p<10−7, paired t-test). (B) Scatter plot showing that the correlation between the decrease in impact factor caused by lag correction (in %) and the duration of the publication lag remains strong after removal of these outliers (r = 0.91, p<10−11). (C) Scatter plot showing the % difference in impact factor caused by lag correction for each journal, both before (x axis) and after (y axis) exclusion of outliers. There is a slight trend for decrease in the lag-corrected difference after outliers are removed, but this does not reach statistical significance (p = 0.12, paired t-test). (TIF) [file pone.0053374.s001.tif]

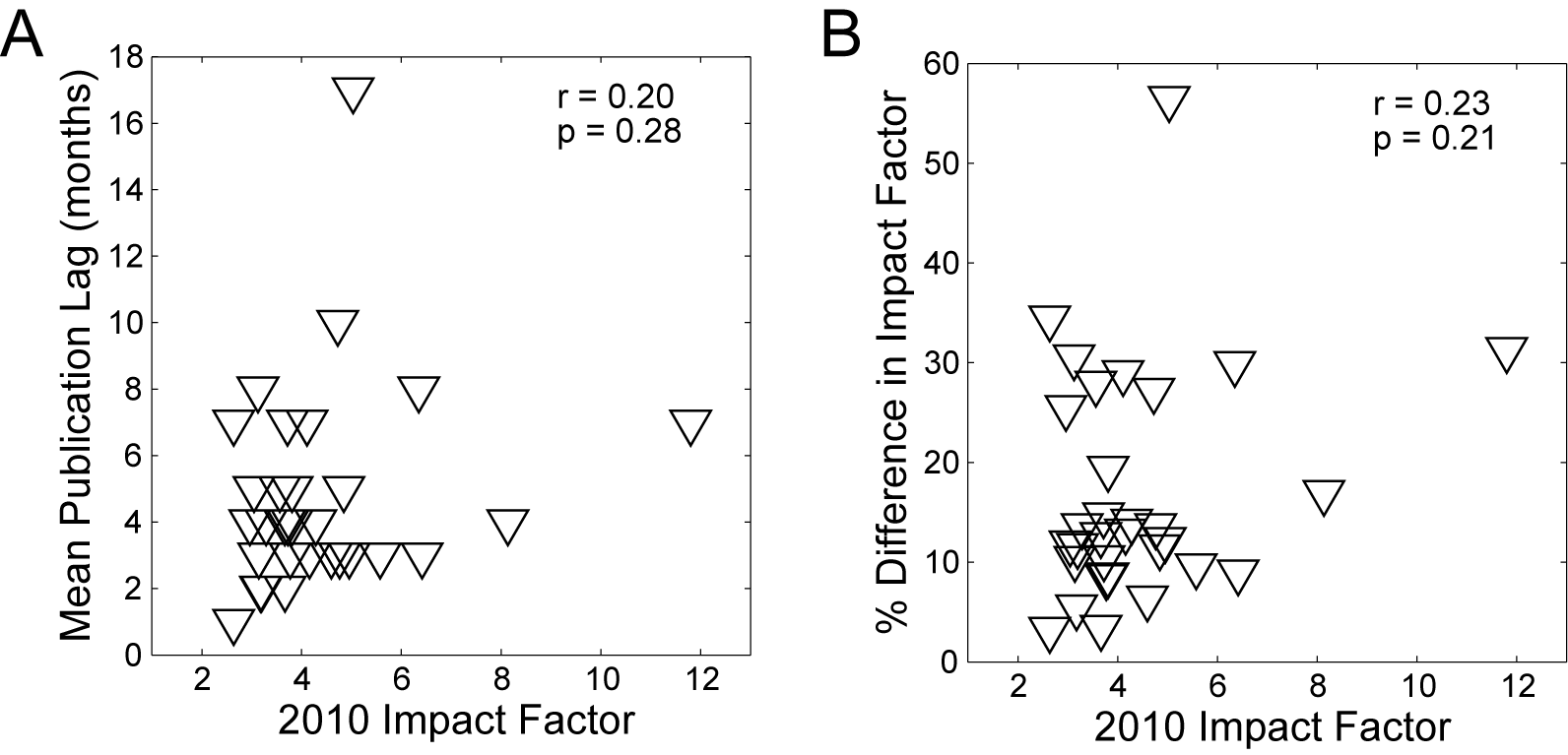

Supplement: Figure S2 — Online-to-print publication lags and inflation of impact factors occur independently of the original journal impact factor. (A) Scatter plot showing absence of significant correlation between journal impact factor and mean publication lag for the 31 journals with lags longer than 3 months (r = 0.20, p = 0.28). (B) Scatter plot showing absence of significant correlation between journal impact factor and relative difference in impact factor after lag correction (r = 0.23, p = 0.21). (TIF) [file pone.0053374.s002.tif]
