# Supplementary material for: Rising Publication Delays Inflate Journal Impact Factors
Source: PLoS One. 2012 Dec 31;7(12):e53374. doi: 10.1371/journal.pone.0053374 (PMC3534064; doi:10.1371/journal.pone.0053374)
Supplement: Table S1 — Journal list. (PDF) [file pone.0053374.s003.pdf]

Supplementary Table S1

Acta Neuropathologica	Annals of Neurology	Behavioural Brain Research
BMC Neuroscience	Biological Psychiatry	Bipolar Disorders
Brain	Brain and Language	Brain Behavior and Immunity
Cephalalgia	Cerebral Cortex	Cortex
European Journal of Neurology	European Journal of Neuroscience	European Journal of Pain
European Neuropsychopharmacology	Experimental Neurology	Genes Brain and Behavior
Glia	Hippocampus	Human Brain Mapping
International Journal of Neuropsychopharmacology	Journal of Cerebral Blood Flow and Metabolism	Journal of Cognitive Neuroscience
Journal of Comparative Neurology	Journal of Neurochemistry	Journal of Neuroendocrinology
Journal of Neuroinflammation	Journal of Neuropathology and Experimental Neurology	Journal of Neurophysiology
Journal of Neuroscience	Journal of Neurotrauma	Journal of Pain
Journal of Physiology London	Journal of Pineal Research	Journal of the Peripheral Nervous System
Journal of Psychopharmacology	Learning and Memory	Molecular and Cellular Neuroscience
Molecular Pain	Molecular Psychiatry	Nature Neuroscience
Nature Reviews Neuroscience	Neurobiology of Aging	Neurobiology of Disease
Neurobiology of Learning and Memory	Neurochemistry International	Neurogastroenterology and Motiliy
Neuroimage	Neuron	Neuropharmacology
Neuropsychopharmacology	Neuroscience	Neurotoxicity Research
Pain	Progress in Neurobiology	Psychoneuroendocrinology
Psychopharmacology	Sleep	Trends in Cognitive Sciences
Trends in Neurosciences		
