# Supplementary material for: Rising Publication Delays Inflate Journal Impact Factors
Source: PLoS One. 2012 Dec 31;7(12):e53374. doi: 10.1371/journal.pone.0053374 (PMC3534064; doi:10.1371/journal.pone.0053374)
Supplement: Table S2 — Lag-corrected impact factors and journal rankings. (PDF) [file pone.0053374.s004.pdf]

Supplementary Table S2

	IF 2010[1]	Ranking[2]	IF 2010 Corrected[3]	Ranking Corrected	Position Change
Behavioural Brain Research	3.29	47	2.84	48	-1
Biological Psychiatry	8.13	8	6.75	11	-3
Brain Behavior and Immunity	3.81	34	3.07	45	-11
Brain and Language	3.05	54	2.68	52	2
Cerebral Cortex	6.35	14	4.45	20	-6
Cortex	6.41	13	5.84	13	0
European Journal of Neurology	3.17	49	3.00	46	3
European Neuropsychopharmacology	3.73	37	3.34	39	-2
European Journal of Pain	3.12	51	2.17	58	-7
Experimental Neurology	4.16	30	3.62	31	-1
Genes Brain and Behavior	2.96	55	2.21	56	-1
Glia	4.85	20	4.29	24	-4
Hippocampus	3.81	35	3.48	35	0
Human Brain Mapping	4.73	22	3.44	37	-15
International Journal of Neuropsychopharmacology	3.72	38	3.17	42	-4
Journal of Cerebral Blood Flow and Metabolism	4.28	28	3.68	29	-1
Journal of Cognitive Neuroscience	4.11	31	2.92	47	-16
Journal of Comparative Neurology	3.67	39	3.54	32	7
Journal of Neurotrauma	3.20	48	2.83	49	-1
Journal of Pineal Research	5.57	15	5.04	15	0
Journal of Psychopharmacology	2.64	57	1.73	60	-3
Molecular and Cellular Neuroscience	3.77	36	3.45	36	0
Molecular Psychiatry	11.80	5	8.11	7	-2
Neurobiology of Aging	5.03	17	2.19	57	-40
Neurobiology of Disease	4.95	18	4.35	22	-4
Neurobiology of Learning and Memory	3.56	42	2.57	53	-11
Neuroimage	4.76	21	4.11	26	-5
Neuropharmacology	3.14	50	2.82	50	0
Neurotoxicity Research	2.64	58	2.55	54	4
Psychoneuroendocrinology	4.59	24	4.30	23	1
Psychopharmacology	3.66	40	3.20	41	-1

[1]The impact factor (IF) shown here is based on counting citations to individual articles, which provides a lower estimate than that reported by ISI (which counts total citations to a journal; see Methods). [2]This ranking refers to 61 neuroscience journals; see Supplementary Table S1 for journal list. [3]The corrected impact factor does not take into account citations occurring during the preprint period (see Main Text for discussion).
